# Supplementary material for: Hydrodynamic manipulation of nano-objects by optically induced thermo-osmotic flows
Source: Nat Commun. 2022 Feb 3;13:656. doi: 10.1038/s41467-022-28212-z (PMC8813924; doi:10.1038/s41467-022-28212-z)
Supplement: Supplementary file 1 — Supplementary Information [file 41467_2022_28212_MOESM1_ESM.pdf]

Supplementary Information

Hydrodynamic Manipulation of Nano-Objects by Optically Induced Thermo-Osmotic Flows

Martin Fränzl[1] **and Frank Cichos**[1,*]

[1] *Peter Debye Institute for Soft Matter Physics, Molecular Nanophotonics Group, Universität Leipzig, Linnéstr. 5, 04103 Leipzig, Germany.*

[*] *cichos@physik.uni-leipzig.de*

Contents

1 Experimental Setup 2

2 Particle Tracking 2

3 Mean-Squared Displacement Analysis 3

4 DLVO Theory 3

5 Escape From DLVO Potential 7

6 Diffusion Close to Surfaces 8

7 Numerical Temperature Simulation 9

8 5CB Temperature Measurement 12

9 Thermo-Osmotic Flow 13

10 Thermophoresis of Colloidal Particles 17

11 Estimation of the Lateral Trap Stiffness 19

12 Optical Forces 20

13 Thermal Convection 21

14 Depletion in Surfactant Solutions 22

1 Experimental Setup

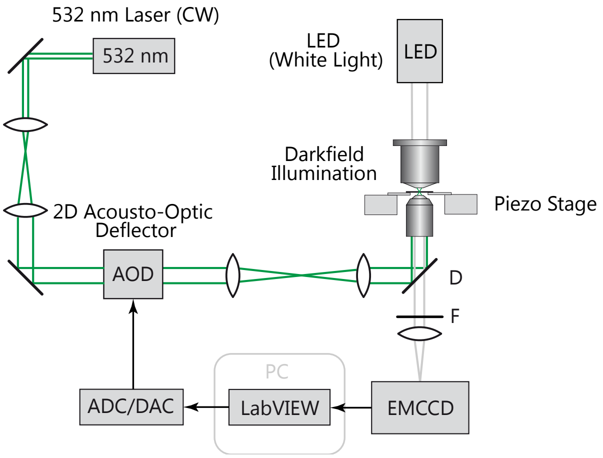

Fig. S1: Sketch of the setup used for the experiments.

2 Particle Tracking

The main steps to detect the particle positions from a recorded image (Fig. S2a) are as follows. First, we reduce the noise in the image using a two pixel median filter and then compute a binary image using a global threshold. Subsequently, a connected-component labeling algorithm is used to identify and filter connected regions according to their pixel area (Fig. S2b). The particles center position is then calculated from the center of mass of the unweighted pixel area and stored along with additional parameters such as the maximum intensity detected within that area. Finally, the particle positions are connected into trajectories using a linking algorithm[1,2]. The z-position of the particle is estimated from the defocusing of the particle[3]. To find a relation between the detected particle radius r_0 and its z-position we performed a reference measurement where we detect r_0 of a particle fixed to the gold film ($z = 0$) and measure the change in z-position using the piezo stage (Fig. S2c).

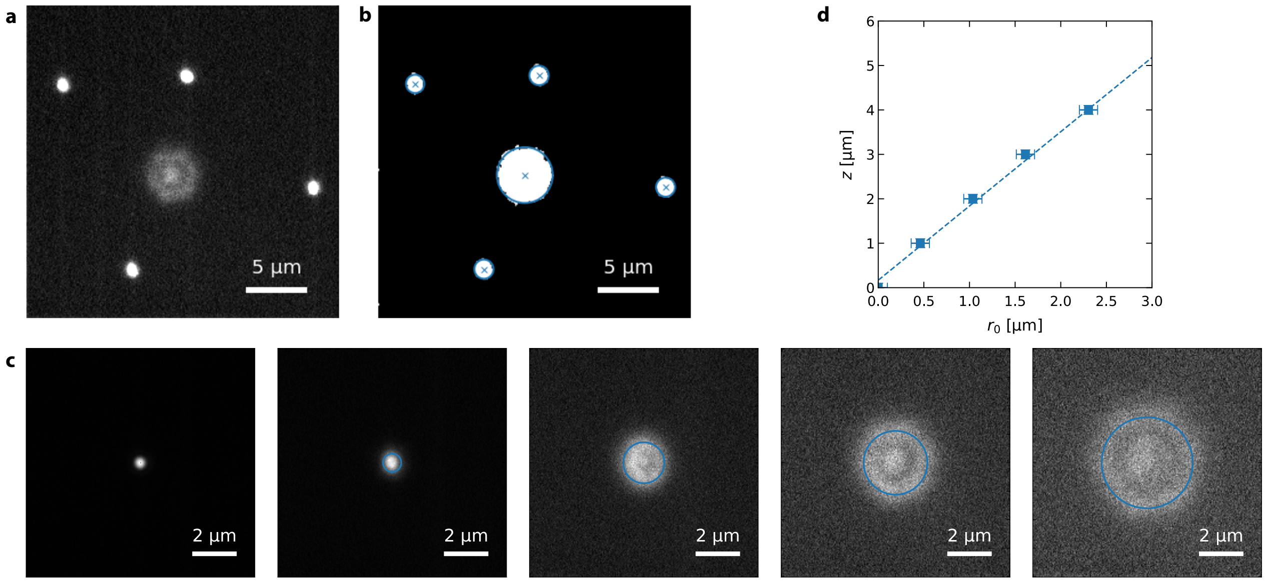

Fig. S2: **a**, The recorded image. **b**, The binary representation of the image processed with a two pixel median filter and a global threshold value. The detected particle centers and radii from the connected-compoment labeling are depicted in blue. **c**, Defocusing of a particle fixed to the gold film. The smallest particle radius is defined as $z = 0$ and the change in z-position is measured using the piezo stage. **d**, The relation between the particle's z-position and its detected particle radius r_0.

3 Mean-Squared Displacement Analysis

The mean squared displacement (MSD) is measured from the ensemble average:

$$\left\langle \Delta r^2(\tau) \right\rangle = \frac{1}{N} \sum_{n=0}^{N} \left(\boldsymbol{r}_n(\tau) - \boldsymbol{r}_n(0) \right)^2 \ , \tag{S1}$$

where N is the number of particle trajectories, $r_n(t)$ the position of the n-th particle at time t and τ the lag time. For a diffusive motion parallel to an interface the MSD is related to the in-plane diffusion coefficient $D_\parallel$ via $\left\langle \Delta r^2(\tau) \right\rangle = 4D_\parallel \tau$. Fig. S3 depicts the measured MSD of gold nanoparticles (NPs) with 250 nm diameter over a 50 nm gold film for different NaCl concentrations c_0.

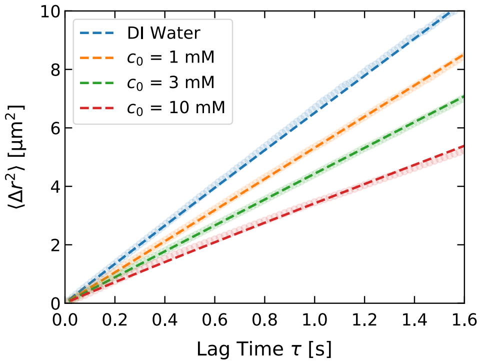

Fig. S3: The mean squared displacement of gold NPs with 250 nm diameter over a 50 nm gold film for different NaCl concentrations c_0 calculated from Eq. (S1). The dashed lines represent linear fits to $\left\langle \Delta r^2(\tau) \right\rangle = 4D_\parallel \tau$. The obtained in-plane diffusion coefficient $D_\parallel$ as function of the NaCl concentration c_0 are shown in Fig. 2a of the main text.

The corresponding in-plane diffusion coefficients $D_\parallel$ are obtained from linear fits to the experimental data (Fig. S3, dashed lines) and plotted as function of the NaCl concentration in Fig. 2a of the main text. We find a decreasing diffusive motion with increasing NaCl concentration.

4 DLVO Theory

The DLVO theory, named after Derjaguin, Landau, Verwey and Overbeek, describes the interaction between charged surfaces in liquid media. The interaction potential comprises an attractive van der Waals contribution V_{wdW} and repulsive electrostatic contribution, V_{E}, due to electric double layer forces:

$$V(d, c_0) = V_{\mathrm{E}}(d, c_0) + V_{\mathrm{vdW}}(d) \ . \tag{S2}$$

Here, d denotes the distance of the surfaces and c_0 the electrolyte concentration in the liquid. The electrostatic potential between the a spherical particle and a plane surface is[4]

$$V_{\mathrm{E}}(d, c_0) \approx 64\pi\varepsilon R \left(\frac{k_{\mathrm{B}}T}{e} \right)^2 \tanh^2 \left(\frac{e\zeta}{4k_{\mathrm{B}}T} \right) \mathrm{e}^{-d/\lambda_{\mathrm{D}}} \ , \tag{S3}$$

where $\varepsilon = 80\,\varepsilon_0$ is the dielectric constant of water, e the elementary charge, ζ the zeta potential and $\lambda_{\mathrm{D}} = \sqrt{\epsilon k_{\mathrm{B}} T / (2 N_{\mathrm{A}} e^2 c_0)}$ the Debye length with the Avogadro constant N_{A}. Note that the concentration c_0 enters Eq. (S3) via the Debye length λ_{D}. The van der Waals potential between a sphere and a plane is[5]

$$V_{\mathrm{VdW}}(d) = -\frac{A_{\mathrm{H}}}{6} \left(\frac{R}{d} + \frac{R}{2R+d} + \ln \left(\frac{d}{2R+d} \right) \right) \ , \tag{S4}$$

where A_{H} is the Hamaker constant. The total potential for gold nanoparticles (AuNPs) with radius $R = 125$ nm as function of the distance d to a Au surface is shown in Fig. S4 for a concentration of $c_0 = 10$ mM. We have used a zeta potential of $\zeta \approx -35$ mV and a Hamaker constant of $A_{\mathrm{H}} = 5 \cdot 10^{-20}$ J.[6]

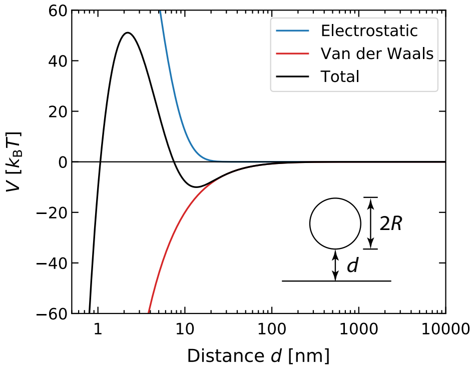

Fig. S4: Plot of the electrostatic and van der Waals contribution to the DLVO potential for a AuNP as function of the distance d to a Au surface for NP radius of $R = 125$ nm and a concentration of $c_0 = 10$ mM. Here, we have used $\zeta = -35$ mV for the zeta potential of the AuNP and the Au surface, respectively, and $A_\mathrm{H} = 5 \cdot 10^{-20}$ J for the Hamaker constant.

Fig. S4 reveals a secondary minimum at $d_\mathrm{min} = 15$ nm. For a particle to get stuck to the surface (primary minimum at $d \sim 0.1$ nm) it needs the overcome the potential barrier between the primary and secondary minimum. Hence, at intermediate concentrations the AuNPs can be confined in the vertical direction, *i.e.*, within the secondary minimum, but are still free to move in the horizontal direction. An additional contribution is represented by the gravitation potential:

$$V_\mathrm{G}(d) = \frac{4}{3}\pi R^3 g\,(\varrho_2 - \varrho_1)\,d\ . \tag{S5}$$

where g is the gravitational constant, ϱ_2 and ϱ_1 the density of the particle and the surrounding liquid, respectively. The gravitation potential gives no significant contribution to the potential at short distances (see Fig. 2d in the main text) but imposes a constant drift velocity $v_\mathrm{G} = -2/(9\eta)R^2 g\,(\varrho_2 - \varrho_1)$ towards the Au surface. For a AuNP with $R = 125$ nm suspended in water we find $v_\mathrm{G} = -0.62\ \mu\mathrm{m\,s^{-1}}$, where we have used $\varrho_1 = 1.0\ \mathrm{kg\,m^{-3}}$, $\varrho_2 = 19.3\ \mathrm{kg\,m^{-3}}$ and $\eta = 0.001$ Pa s.

Concentration Dependence

Fig. S5a, b show the DLVO potential of a AuNP ($R = 125$ nm) as function of d for different concentrations c_0. The depth of the secondary minimum V_min increases linearly with increasing concentration (Fig. S5c) and the minimum location, d_min, is shifted to smaller distances and scales with $d_\mathrm{min} \propto c_0^{-3/4}$ (Fig. S5d). Furthermore, the potential barrier towards the primary minimum decreases with increasing concentration c_0. If c_0 is too high (> 48 mM) the secondary minimum vanishes (Fig. S5a, dashed black line) and the AuNPs will adhere to the Au surface (primary minimum of the DLVO potential).

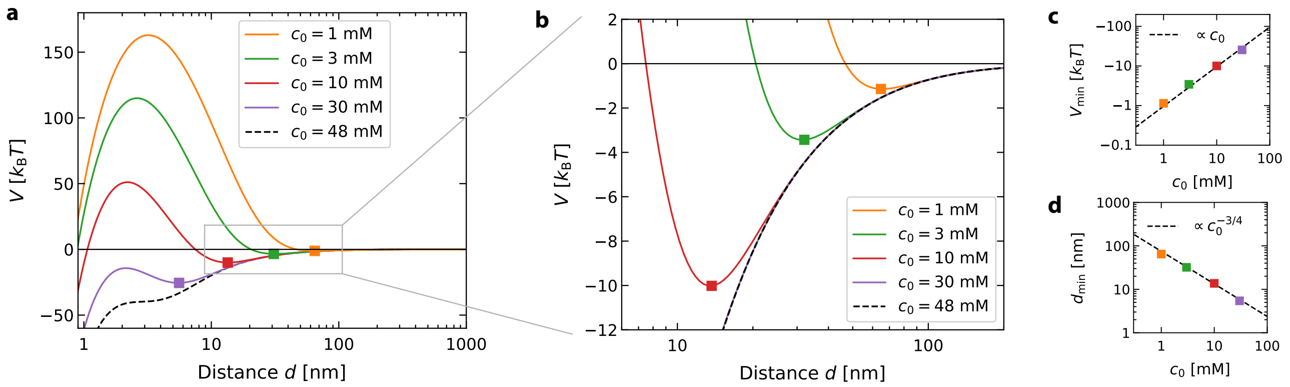

Fig. S5: **a**, Plot of the DLVO potential between a AuNP ($R = 125$ nm) and a Au surface as function of the particle–surface distance d for different concentrations c_0 ($\zeta = -35$ mV, $A_\mathrm{H} = 5 \cdot 10^{-20}$ J). **b**, shows a zoomed-in view of (**a**). **c** and **d** depict the depth V_min and the location d_min of the secondary minimum corresponding to the squares in (**a**) as function of the concentration c_0, respectively.

With respect to the sample preparation (see the Methods section) we realized concentrations down to 10 mM. For concentrations above 10 mM, even though possible in theory, all AuNPs are immobilized at the Au surface as a consequence of the lowered potential barrier towards the primary minimum. During the cover slide assembly the AuNPs attain enough potential energy to overcome the lower potential barrier and adhere to the Au surface.

Size Dependence

The influence of the AuNP radius R on the DLVO potential is depicted in Fig. S6a for a concentration of 10 mM. The depth of the secondary minimum $V_{\min}$ increases with increasing NP radius R and scales with $V_{\min} \propto R^{4/3}$ (Fig. S6b), whereas as the location of the minimum is largely unchanged (Fig. S6, dashed gray line).

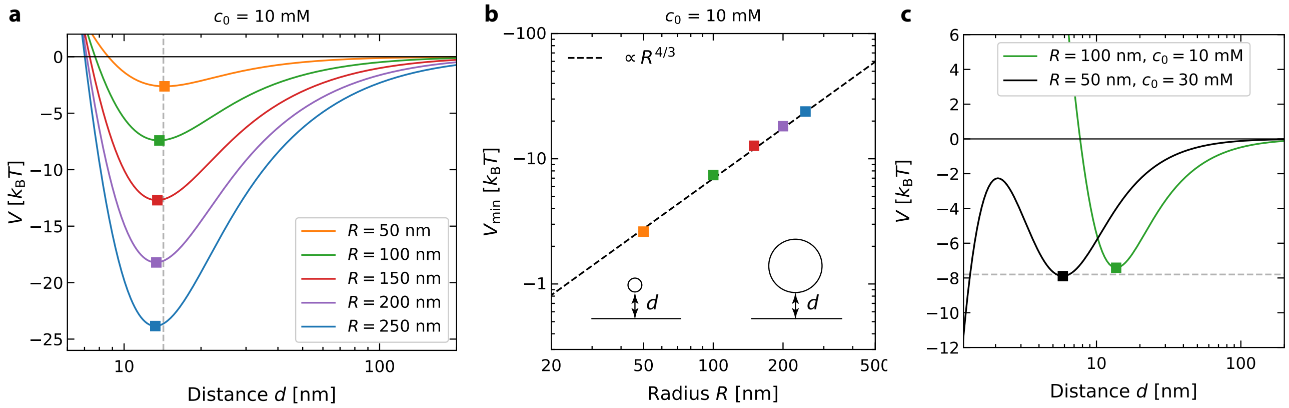

Fig. S6: a, Plot of the DLVO potential between a AuNP and a Au surface as function of the particle–surface distance d for different NP radii R at a concentration of c_0 ($\zeta = -35$ mV, $A_H = 5 \cdot 10^{-20}$ J). **b**, depicts the depth $V_{\min}$ of the secondary minimum corresponding to the squares in (**a**) as function of the NP radius R. **c**, shows the concentration matched potential depth $V_{\min}$ of differently sized NPs.

Given a AuNP with $R = 100$ nm at $c_0 = 10$ mM the same depth of the secondary minimum can be achieved for a smaller NP size ($R = 50$ nm) by increasing the concentration to $c_0 = 30$ mM (Fig. S6c). In view of Fig. S5 the secondary minimum is then observed at a smaller distance $d_{\min}$. The confinement of a NP with $R = 50$ nm at a concentration of 30 mM NaCl is demonstrated in Supplementary Video 5.

Material Dependence

The material dependence of the DLVO potential is defined by the Hamaker constant A_H of the material system and zeta potential ζ of the surfaces. The macroscopic Hamaker constant defines the interaction of medium 1 with medium 2 across medium 3 and a theoretical description is given within the Lifschitz theory of the Hamaker constant.[4] Typically, the Hamaker constant of metals is larger than for dielectric media. The theoretical value for the Au/water/Au system is $10 \cdot 10^{-20}$ J. However, for a thin Au film on a glass surface slightly lower values of the Hamaker constant are expected.[6] We have used $A_H = 5 \cdot 10^{-20}$ J. In case of polystyrene (PS) NPs we have employed $A_H = 1 \cdot 10^{-20}$ J for the PS/water/Au system. The corresponding DLVO potential is plotted in Fig. S7 for a PS NP ($R = 125$ nm) with a zeta potential of $\zeta = -35$ mV to allow for a direct comparison with Fig. S5b. For a given concentration, *e.g.*, 10 mM, the secondary minimum is smaller in comparison to the AuNP system. Remarkably, with increasing concentration large values of $-V_{\min}$ can be achieved before the secondary minimum vanishes at about 1 M.

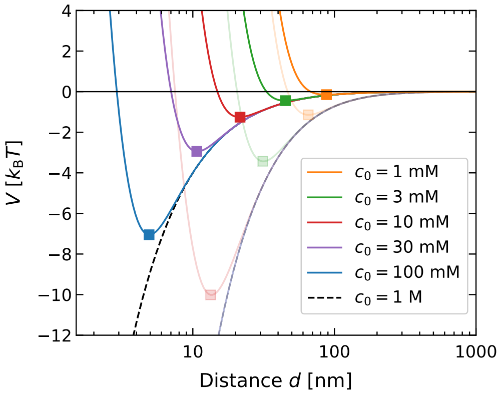

Fig. S7: Plot of the DLVO potential between a PS NP (R = 125 nm) and a Au surface as function of the particle–surface distance d for different concentrations c_0 ($\zeta = -35$ mV, $A_H = 5 \cdot 10^{-20}$ J). The graphs in the background depicts the corresponding potentials for a AuNP of the same size (Fig. S5b).

Mean Surface-to-Surface Distance

The probability to find a particle at a given z-position in the sample is given by the Boltzmann distribution:

$$p(d) = p_0 \, \mathrm{e}^{-V(d,c_0)/(k_B T)} \ , \tag{S6}$$

where $d = z - R$ is the surface-to-surface distance, $V(d, c_0)$ is the DLVO potential given in Eq. (S2). The normalizing constant p_0 is defined as:

$$p_0 \int_0^{H-R} \mathrm{e}^{-V(d,c_0)/(k_B T)} \, \mathrm{d}d = 1 \ , \tag{S7}$$

where the upper limit of the integration is given by the sample height H minus the particle radius R. The mean surface-to-surface distance $\langle d \rangle$ is then defined by:

$$\langle d \rangle = p_0 \int_0^{H-R} d \, \mathrm{e}^{-V(d,c_0)/(k_B T)} \, \mathrm{d}d \ . \tag{S8}$$

Fig. S8 depicts $\langle d \rangle$ as function of the concentration c_0 for a sample height of $H = 3$ µm.

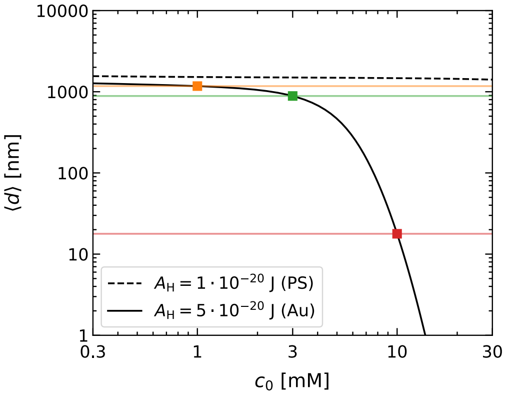

Fig. S8: The mean distance $\langle d \rangle$ as function of the concentration c_0 according to Eq. (S8) for a sample height of $H = 3$ µm. The black solid line corresponds to the DLVO potential defined as in Fig. S5 ($A_H = 5 \cdot 10^{-20}$ J), whereas the black dashed line corresponds to a Hamaker constant of $A_H = 1 \cdot 10^{-20}$ J.

Similar, the mean in-plane diffusion coefficient is defined by:

$$\langle D_\parallel \rangle = p_0 \int_0^{H-R} D_\parallel(d) \, \mathrm{e}^{-V(d,c_0)/(k_B T)} \, \mathrm{d}d \ . \tag{S9}$$

where $D_\parallel(d)$ denotes the distance-dependent in-plane diffusion coefficient given in Eq. (S9).

Force Calculation

The force exerted on the NP is readily obtained from:

$$F = -\frac{\partial V}{\partial d} \ . \tag{S10}$$

Fig. S9a depicts the DLVO potential for a AuNP with $R = 125$ nm radius at $c_0 = 10$ mM. The corresponding force calculation is shown in Fig. S9b. At a distance of 20 nm we obtained a maximum force of -1.6 pN (see Fig. 3a in the main text). The DLVO potential in the vicinity of the secondary minimum is non-harmonic. A rough approximation with a harmonic potential is shown in Fig. S9a and yields to a vertical trap stiffness k_z in the order of 500 pN μm^{-1}.

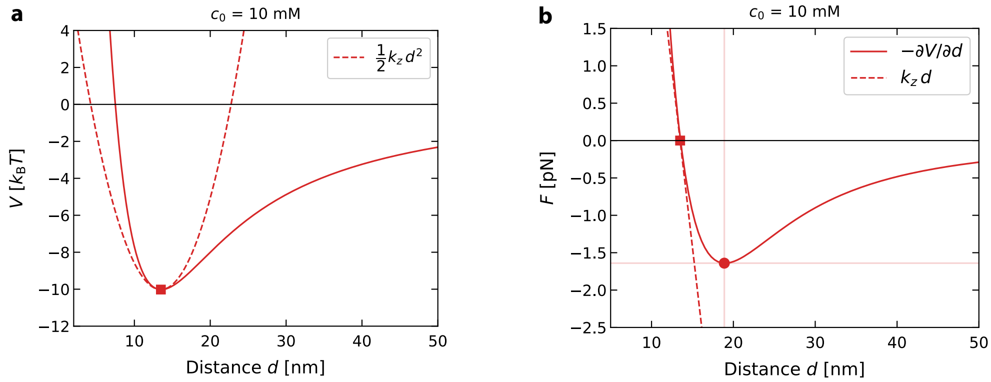

Fig. S9: **a**, The DLVO potential for a AuNP with $R = 125$ nm radius at $c_0 = 10$ mM. **b**, The force corresponding to (**a**) calculated using $F = -\partial V/\partial d$.

5 Escape From DLVO Potential

Considering the Fokker–Planck equation with a distance-dependent diffusion coefficient $D_\perp(d)$ and a potential $V(d)$ the mean escape time is derived as:

$$\langle \tau \rangle = \int_{d_{\min}}^{d_{\max}} \left(\frac{\mathrm{e}^{V(y)/(k_\mathrm{B}T)}}{D_\perp(y)} \int_{-\infty}^{y} \mathrm{e}^{-V(x)/(k_\mathrm{B}T)} \,\mathrm{d}x \right) \mathrm{d}y \ , \tag{S11}$$

where $d_{\min}$ is the location of the secondary minimum, $d_{\max}$ the escape distance and $D_\perp(d)$ the out-off-plane diffusion coefficient (see Eq. (S14) in Sec. 6). Here, $d_{\max}$ defines as the distance where we assume the particle has escaped the potential. Fig. S10 depicts the mean escape time as function of the concentration c_0 for an escape distance of $d_{\max} = 500$ nm calculated from a numerical integration of Eq. (S11).

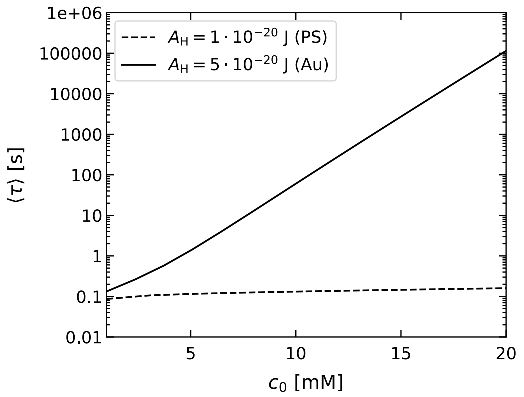

Fig. S10: The mean escape time $\langle \tau \rangle$ as function of the concentration c_0 for an escape distance of $d_{\max} = 500$ nm. The black solid line corresponds to the DLVO potential defined as in Fig. S5 ($A_\mathrm{H} = 5 \cdot 10^{-20}$ J), whereas the black dashed line corresponds to a Hamaker constant of $A_\mathrm{H} = 1 \cdot 10^{-20}$ J.

6 Diffusion Close to Surfaces

The diffusion coefficient of a spherical particle in a bulk liquid is defined by the Stokes–Einstein relation:

$$D_0 = \frac{k_\mathrm{B} T}{f_0} \; , \tag{S12}$$

where k_B is the Boltzmann constant, T the temperature and $f_0 = 6\pi\eta R$ the friction coefficient. Typically, the fiction of a confined particle is increased resulting in a lower diffusion coefficient. In particular, the friction is different for a motion parallel and perpendicular to a surface. The correction factor $\gamma_\parallel$ for the friction coefficient parallel to a surface, $f_\parallel = f_0\gamma_\parallel$, is approximated by:[7]

$$\gamma_\parallel^{-1}(z) = \frac{D_\parallel}{D_0} \approx 1 - \frac{9}{16}\frac{R}{z} + \frac{1}{8}\left(\frac{R}{z}\right)^3 - \frac{45}{256}\left(\frac{R}{z}\right)^4 - \frac{1}{16}\left(\frac{R}{z}\right)^5 \pm \ldots \; , \tag{S13}$$

where z is the distance from the particle center to the surface and R the radius of the particle (Fig. S11a). The correction factor $\gamma_\perp$ for the friction coefficient in perpendicular to a surface, $f_\perp = f_0\gamma_\perp$, is given by the exact solution:[8]

$$\gamma_\perp^{-1}(z) = \frac{D_\perp}{D_0} = \left(\frac{4}{3}\sinh\alpha \sum_{n=1}^{\infty} \frac{n(n+1)}{(2n-1)(2n+3)} \left(\frac{2\sinh((2n+1)\alpha) + (2n+1)\sinh(2\alpha)}{4\sinh^2((n+1/2)\alpha) - (2n+1)^2\sinh^2\alpha} - 1\right)\right)^{-1} \; , \tag{S14}$$

with $\alpha = \operatorname{arcosh}(z/R)$. Fig. S11b depicts the predicted values for $D_\parallel/D_0$ and $D_\perp/D_0$ as function of the relative particle-surface distance z/R.

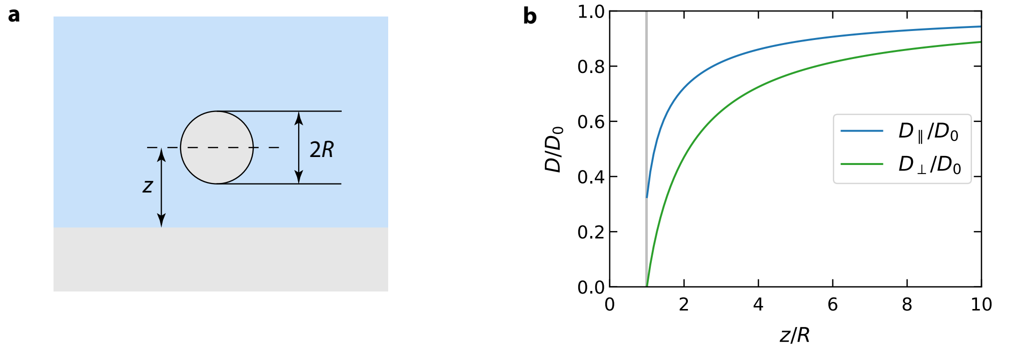

Fig. S11: **a**, Sketch of a spherical particle with radius R located at distance z from a surface. **b**, The values of $D_\parallel/D_0$ and $D_\perp/D_0$ as function of the relative particle-surface distance z/R calculated from Eq. (S13) and Eq. (S14).

If the particle is located between two surfaces with distance H (Fig. S12a) the correction factors for the friction coefficient can be approximated by a linear superposition:

$$\gamma^\mathrm{II}(z) = \gamma^\mathrm{I}(z) + \gamma^\mathrm{I}(H-z) - 1 \; , \tag{S15}$$

where $\gamma^\mathrm{I}(z)$ represents either $\gamma_\parallel(z)$ or $\gamma_\perp(z)$. An even better approximation is be given by:[9]

$$\gamma^\mathrm{II}(z) = 1 + \sum_{n=0}^{\infty}\left(\gamma^\mathrm{I}(z+nH) - 1\right) + \sum_{n=1}^{\infty}\left(\gamma^\mathrm{I}(nH-z) - 1\right) - 2\sum_{n=1}^{\infty}\left(\gamma^\mathrm{I}(nH) - 1\right) \; . \tag{S16}$$

Fig. S12b compares $D_\parallel/D_0$ to $D_\parallel^\mathrm{II}/D_0$ for $R = 125$ nm and $H = 3$ µm. For $H/R \gg 2$ the correction factors are well approximated by the single surface correction.

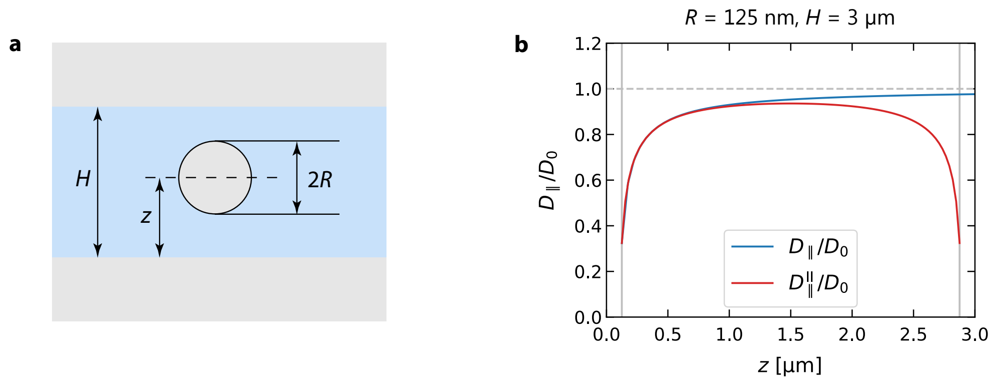

Fig. S12: **a**, Sketch of a spherical particle with radius R located at distance z from a surface and confined by a second surfaces at distance H. **b**, Depicts $D_{\|}/D_0$ and $D_{\|}^{\|\|}/D_0$ for $R = 125$ nm and $H = 3$ µm calculated from Eq. (S13) and Eq. (S16)

7 Numerical Temperature Simulation

The stationary temperature distribution in the sample was deduced from finite-element simulations using the heat transfer module of COMSOL Multipyhsics 5.1. The sample geometry was represented by a 2D axisymmetric model as depicted in the Fig. S13. To minimize boundary effects the simulation geometry was implemented sufficiently large.

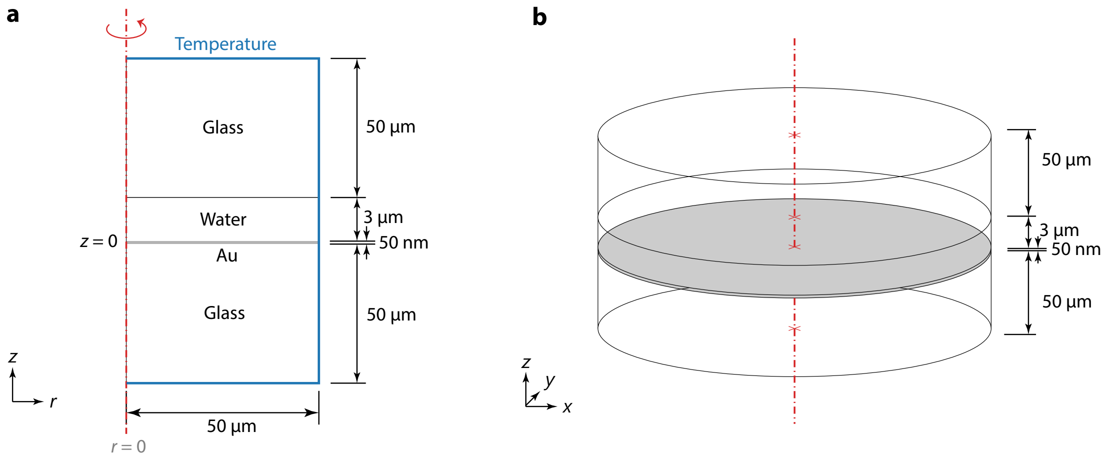

Fig. S13: **a**, Sketch of the 2D axisymmetric model employed to simulate the temperature distribution in the sample using COMSOL Multipyhsics 5.1 (dimensions are not to scale). The heat source domain, *i.e.*, the gold film, is colored in gray. **b**, The revolved simulation geometry in 3D.

In the simulation we set the outer boundary layers to room temperature $T_0 = 293.15$ K and neglected thermal convection in the liquid film due to its small height (see Section 13 for details). To account for the optical absorption of the focused laser beam we defined a heat source within the gold film domain (gray domain) as follows:

$$Q(r,z) = P_0\,(1-R)\,\frac{2\alpha}{\pi w_0^2}\,\mathrm{e}^{-2r^2/w_0^2}\,\mathrm{e}^{-\alpha z}\ , \tag{S17}$$

where P_0 is the incident laser power, w_0 the beam waist of the focused laser, R the reflectance and α absorption coefficient of the gold film at the laser wavelength $\lambda = 532$ nm. A reflectance simulation of a 50 nm gold film as function of the incident wavelength is plotted in Fig. S14. The refractive index of gold was taken from Ref. 10. For $\lambda = 532$ nm we find $R = 0.6$ and an absorption coefficient of $\alpha = 4\pi n''/\lambda = 5.3 \cdot 10^7\ \mathrm{m}^{-1}$, where n'' denotes the imaginary part of the refractive index at the given wavelength.

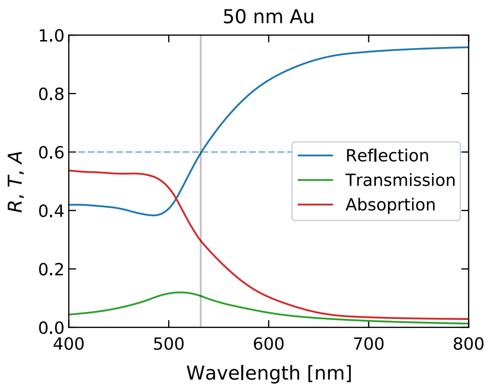

Fig. S14: Simulated transmittance T, reflectance R and absorptance A of a 50 nm gold film. The refractive index of gold was taken from Ref. 10.

For the thermal conductivity κ of the gold film we have used $\approx 150\ \mathrm{W\,m^{-1}\,K^{-1}}$ according to Ref. 11, whereas for the other material properties we have taken the bulk values (Tab. S1). Note that only the thermal conductivity determines the stationary solution of the heat equation.

Tab. S1: The material properties used in the COMSOL simulation, where κ is the thermal conductivity, ρ the density and c_p the heat capacity at constant pressure.

Material	κ [$\mathrm{W\,m^{-1}\,K^{-1}}$]	ϱ [$\mathrm{g\,cm^{-3}}$]	c_p [$\mathrm{J\,kg^{-1}\,K^{-1}}$]
Glass (BK7)	1.2	2.2	830
Au	150	19.3	129
Water	0.6	1.0	4182

Fig. S15 depicts the simulated temperature distribution for an incident laser power $P_0 = 1$ mW. We obtain a maximum temperature increment at the heat spot of about $\Delta T_{\mathrm{max}} = 25$ K. The related temperature gradient is plotted in Fig. S16. For very thin water films the maximum temperature increment is expected to decrease due to the larger thermal conductivity of glass compared to water.

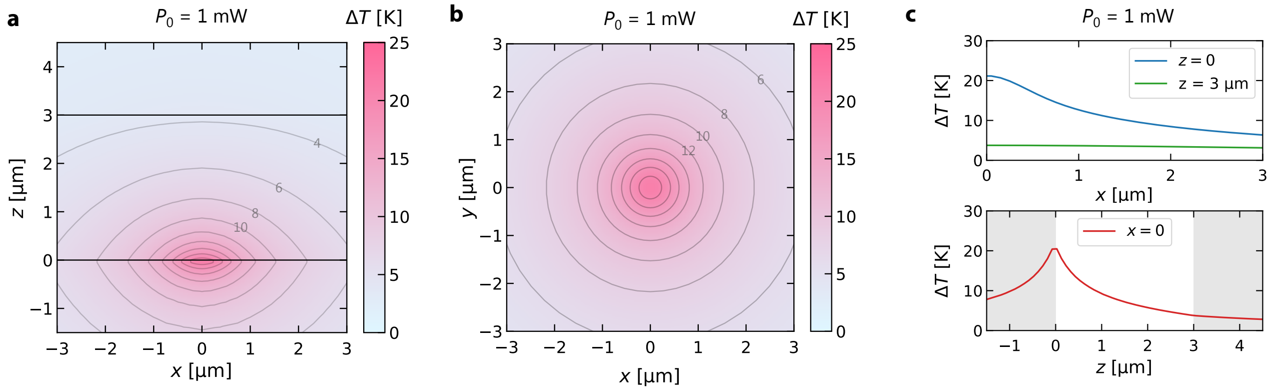

Fig. S15: Simulated temperature distribution for an incident laser power of $P_0 = 1$ mW. **a**, Cross-section of temperature distribution in the xz-plane at $y = 0$. **b**, Top view of the temperature distribution in the xy-plane at $z = 0$. **c**, Radial temperature profile in x-direction for $y = 0$ at different heights ($z = 0, 5$ μm) in the liquid film. **d**, Vertical temperature profile in z-direction at $x, y = 0$.

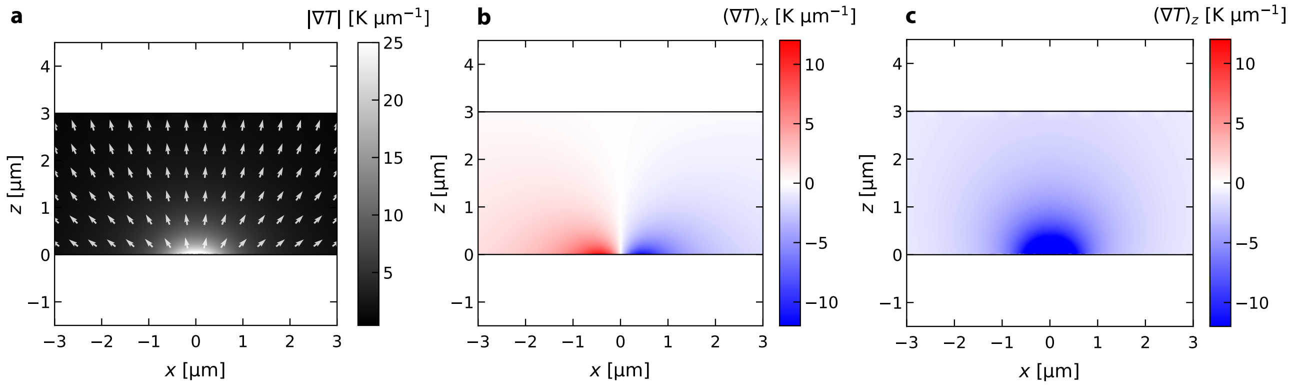

Fig. S16: The temperature gradient calculated from Fig. S15a. **a**, The magnitude of the temperature gradient and its direction (arrows) in the xz-plane ($y = 0$). **b**, x-component of the temperature gradient shown in (**a**). **c**, z-component of the temperature gradient in (**a**).

The maximum temperature increment ΔT_{max} as function of the incident laser power is plotted in Fig. S17 and reveals a linear dependence, $\Delta T_{\mathrm{max}} \propto P_0$.

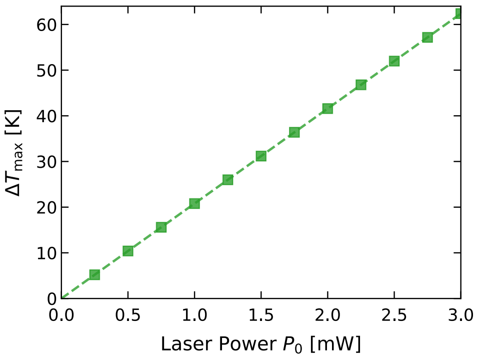

Fig. S17: Simulated maximum temperature increment ΔT_{max} as function of the incident laser power P_0.

8 5CB Temperature Measurement

To estimate the temperature increment on the gold film a method based on the phase transition of the liquid crystal 5CB is used. Therefore, the gold film was embedded in a 5CB sample and heated with a focused laser as illustrated in Fig. S18a. The phase transition of 5CB occurs at $T_{pt} = 35\,°C$. For $T < 35\,°C$ the liquid crystal is in its nematic phase, where the molecules are stacked and oriented in a preferred direction. For $T > 35\,°C$ the order vanishes and the liquid crystal is in its isotropic phase.[12] Hence, a local isotropic phase around a heated spot occurs whenever the local temperature exceeds the phase transition temperature T_{pt}. Due to the large refractive index difference between the isotropic phase and the surrounding nematic phase, the radius of the phase transition R_{pt} can be easily determined using a dark-field optical microscope. The radius of the phase transition is expected to increase linearly with the incident laser power since $\Delta T \propto P_0$. The blue squares in Fig. S18b depict the obtained phase transition radius R_{pt} as function of the incident laser power P_0 for a gold film thickness of 50 nm. The corresponding dark-field optical microscopy images are shown in Fig. S18c. The temperature increment in 5CB is then given by:

$$\Delta T^{5CB}_{max} = \frac{(35\,°C - T_0)}{P_0^{pt}} P_0 \,, \tag{S18}$$

where $T_0 = 25\,°C$ is the ambient temperature and P_0^{pt} the power where the phase transition temperature $T_{pt} = 35\,°C$ is first reached. We find $P_0^{pt} = 0.48$ mW from a from a linear fit (blue dashed line) in Fig. S18.

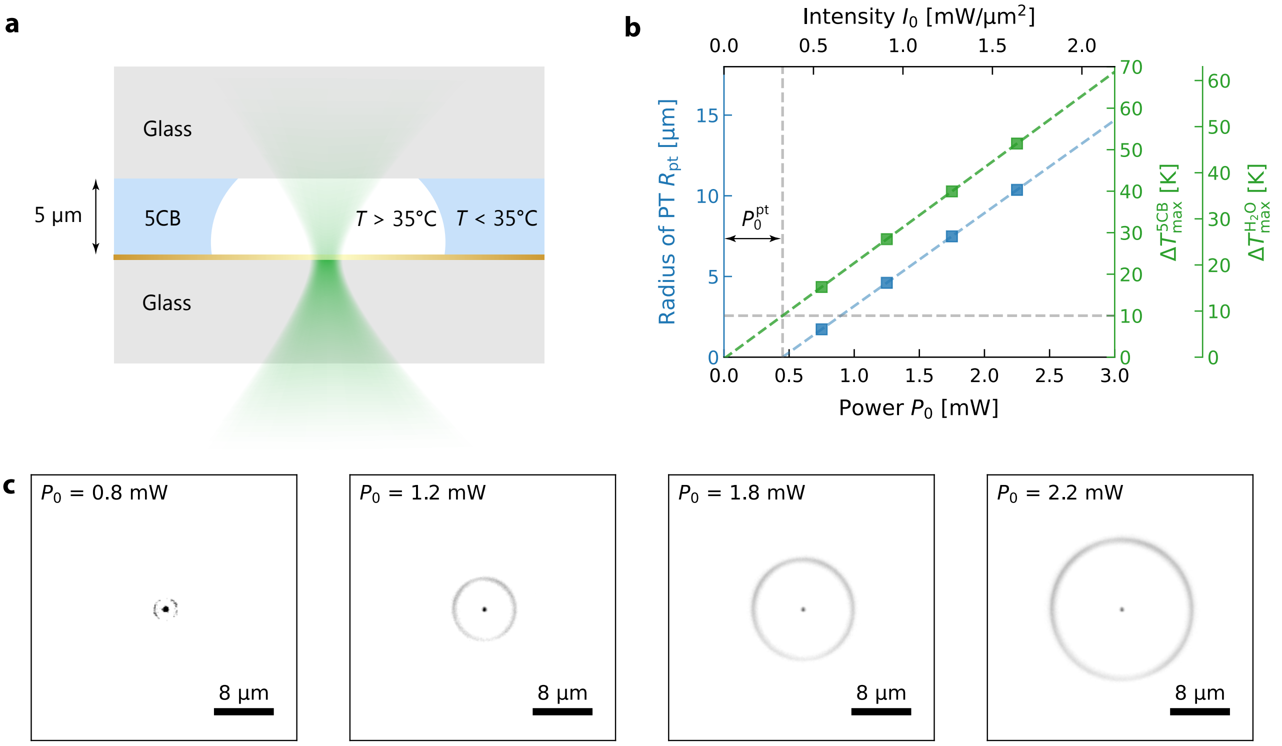

Fig. S18: Temperature Measurement using 5CB. a, Sketch of the measurement principle and the sample geometry. **b**, The measured phase transition radius (R_{pt}, blue squares) and the temperature increment in 5CB (ΔT^{5CB}_{max}, green squares) calculated using Eq. (S18) as functions of the incident laser power P_0. The temperature increment in water ($\Delta T^{H_2O}_{max}$) is by a factor of 0.9 smaller than in 5CB according to the numerical simulation in Section 7. **c**, The darkfield optical microscopy images corresponding to the data points in (**b**).

The temperature increment in water is calculated as $\Delta T^{H_2O}_{max} = 0.9\,\Delta T^{5CB}_{max}$, where the factor 0.9 accounts for the higher thermal conductivity of water and is obtained from a numerical simulation (see Section 7 for details). The thermal conductivity of 5CB in its isotropic phase is about $\kappa_{5CB} = 0.15\ W\,m^{-1}\,K^{-1}$.[13] The thermal conductivity of water is about $\kappa_{H_2O} = 0.6\ W\,m^{-1}\,K^{-1}$. Hence, the temperature increment of the gold film is expected to be smaller when embedded in water. The finally obtained temperature increment ($\Delta T^{H_2O}_{max}$, Fig. S18b) agrees well with the simulation result in Fig. S17.

9 Thermo-Osmotic Flow

A temperature gradient along the liquid–solid interfaces in will cause a slip flow in the liquid boundary layer. If we take a liquid volume element close to the solid from the cold side and exchange that with one at the hot side would not only transport heat, since the liquid volumes have different temperature, but also additional free energy as the liquid has a different interaction with the solid in these regions. This excess enthalpy is causing a liquid flow at the interface, which is called thermo-osmotic flow. Since the interaction range is small, *i.e.*, only a few nanometers, the hydrodynamics can be treated in a boundary layer approximation.

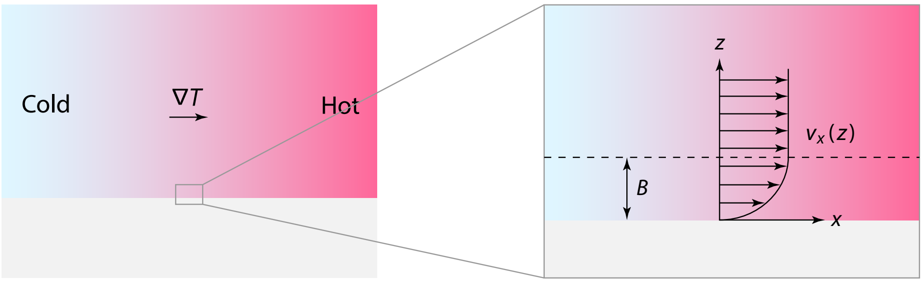

Fig. S19: Sketch of the boundary layer approximation. Due to the liquid–solid interactions the fluid velocity parallel to the solid surface v_x increases in z-direction and gets constant at distances beyond the interaction range B.

Boundary Layer Approximation

The fluid flow in the micro- and nanometer scale is governed by low Reynolds number hydrodynamics. Hence, for an incompressible fluid ($\nabla \cdot \boldsymbol{v} = 0$), the velocity field $\boldsymbol{v}$ is defined by the Stokes equation:

$$\eta \nabla^2 \boldsymbol{v} - \nabla P + \boldsymbol{f} = 0 \,, \tag{S19}$$

where P is the pressure, η the viscosity and $\boldsymbol{f}$ the force density in the fluid. We consider a solid/liquid interface and local coordinates as shown in Fig. S19. Close to the interface the vertical component of the velocity vanishes, $v_z = 0$. Since the hydrodynamic quantities vary much more rapidly in the vertical direction than parallel to the surface we approximate $v_x(x, z) \approx v_x(z)$ and the Stokes equation can be reduced to:

$$0 = \frac{\mathrm{d}P}{\mathrm{d}z} - f_z \,, \tag{S20}$$

$$\eta \frac{\mathrm{d}^2 v_x}{\mathrm{d}z^2} = \frac{\mathrm{d}P}{\mathrm{d}x} - f_x \,. \tag{S21}$$

The velocity is zero at the solid surface, $v_x|_{z=0} = 0$, and takes a constant value at infinity, $v_\parallel = v_x(\infty)$. Since $v_x|_{z=0} = 0$, we can write:

$$v_x(z) = \int_0^z \frac{\mathrm{d}v_x(z')}{\mathrm{d}z'}\, \mathrm{d}z' = v_x(z')|_{z'=z} - \underbrace{v_x(z')|_{z'=0}}_{=0} \tag{S22}$$

Hence, the boundary velocity reads:

$$v_\parallel = v_x(\infty) = \int_0^\infty 1 \cdot \frac{\mathrm{d}v_x(z')}{\mathrm{d}z'}\, \mathrm{d}z' = \underbrace{z' \left.\frac{\mathrm{d}v_x(z')}{\mathrm{d}z'}\right|_0^\infty}_{=0} - \int_0^\infty z' \frac{\mathrm{d}^2 v_x(z')}{\mathrm{d}z'^2}\, \mathrm{d}z' \,, \tag{S23}$$

where we have used integration in parts. Since, $\mathrm{d}v_x/\mathrm{d}z|_{z=\infty} = 0$, the first term on the right hand side vanishes and we find:

$$v_\parallel = -\frac{1}{\eta} \int_0^\infty z \left(\frac{\mathrm{d}P}{\mathrm{d}x} - f_x \right) \mathrm{d}z \,, \tag{S24}$$

where we have inserted Eq. (S21) and replaced z' with z. Eq. (S24) can be generalized in terms of a local excess enthalpy $h(z)$ that accounts for the specific interactions between the liquid and the solid:

$$v_\parallel = -\frac{1}{\eta} \int_0^\infty z\, h(z)\, \mathrm{d}z\, \frac{\nabla_\parallel T}{T} \,, \tag{S25}$$

where $\nabla_{\|}T$ is the temperature gradient parallel to the surface. Furthermore, Eq. (S25) can be condensed to:

$$v_{\|} = \chi \, \frac{\nabla_{\|}T}{T} \; , \tag{S26}$$

where all information on the interfacial interactions is summarized in a thermo-osmotic coefficient χ. If $\chi < 0$ the the liquid is driven to the cold, whereas for $\chi > 0$, the liquid is driven to the hot.

Electric Double Layer Contribution

We consider the electric double layer of a charged liquid–solid interface (Fig. S20). In the mean field approximation the excess densities of (monovalent) positive and negative ions are given by $n_{\pm} = n_0 \left(\exp\left(\mp e\psi/k_{\mathrm{B}}T\right) - 1\right)$, where n_0 is the bulk salinity. Hence, for the charge density σ and the excess ion density n we get:

$$\varrho = e\left(n_+ - n_-\right) = -2en_0 \sinh(e\psi/k_{\mathrm{B}}T) \; , \tag{S27}$$
$$n = n_+ + n_- = 2n_0(\cosh(e\psi/k_{\mathrm{B}}T) - 1) \; . \tag{S28}$$

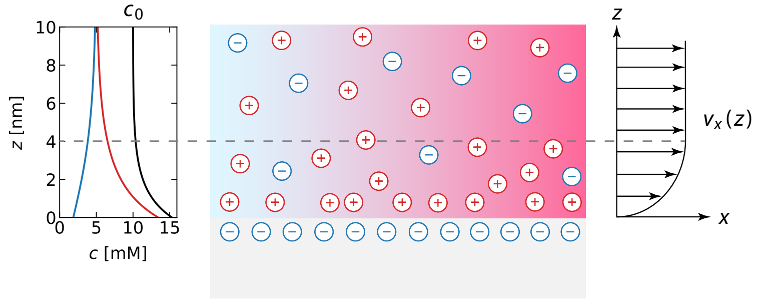

Fig. S20: The electric double layer close to a negatively charged surface. The graph on the left-hand side depicts the concentration of positive (red) and negative ions (blue) as function of the distance to the surface for a bulk concentration of $c_0 = 10$ mM. The black graph represents the total ion concentration. The pressure in the boundary layer is slightly higher at the cold side and drives the charged liquid to the warm; the resulting velocity profile reaches its maximum value beyond the screening length, which is indicated by the dashed line.

The force $\boldsymbol{f}\mathrm{d}V$ exerted on a volume element $\mathrm{d}V$ of a fluid is:

$$\boldsymbol{f} = \nabla \cdot \mathcal{T} + \varrho \boldsymbol{E}_0 \; , \tag{S29}$$

where $\mathcal{T}$ is the Maxwell stress tensor and the first term, $\nabla \cdot \mathcal{T} = \varrho \boldsymbol{E} - \frac{1}{2}E^2\nabla\varepsilon$, comprises the electric force exerted by the charged surface on the liquid. The second term accounts for the interaction of the liquid with an external electric field $\boldsymbol{E}_0$. With the electric field $\boldsymbol{E} = -\nabla\psi$ the force density reads:

$$\boldsymbol{f} = -\varrho\nabla\psi - \frac{1}{2}E^2\nabla\varepsilon + \varrho\boldsymbol{E}_0 \; . \tag{S30}$$

For the osmotic pressure, $P = nk_{\mathrm{B}}T$, we find:

$$\nabla P = \varrho\nabla\psi + (\varrho\psi + nk_{\mathrm{B}}T)\frac{\nabla T}{T} + nk_{\mathrm{B}}T\frac{\nabla n_0}{n_0} \; ,$$

where we have used Eq. (S27) and (S28). Together, for the right-hand side of Stokes' equation we get:

$$\boldsymbol{f} - \nabla P = -(\varrho\psi + nk_{\mathrm{B}}T)\frac{\nabla T}{T} - \frac{1}{2}E^2\nabla\varepsilon - nk_{\mathrm{B}}T\frac{\nabla n_0}{n_0} + \varrho\boldsymbol{E}_0 \; . \tag{S31}$$

Note that the terms $\varrho\nabla\psi$ have canceled out. In the Debye–Hückel approximation we have:

$$\varrho \approx -\frac{\varepsilon}{\lambda_{\mathrm{D}}^2}\psi \; , \qquad nk_{\mathrm{B}}T \approx \frac{\varepsilon}{2\lambda_{\mathrm{D}}^2}\psi^2 \; , \qquad E \approx \frac{1}{\lambda_{\mathrm{D}}}\psi \; ,$$

with $\psi = \zeta \mathrm{e}^{-z/\lambda_\mathrm{D}}$ and for Eq. (S31) we find:

$$\boldsymbol{f} - \nabla P \approx \left(\frac{\varepsilon}{\lambda_\mathrm{D}^2}\psi^2 - \frac{\varepsilon}{2\lambda_\mathrm{D}^2}\psi^2 \right) \frac{\nabla T}{T} - \frac{1}{2\lambda_\mathrm{D}^2}\psi^2 \nabla\varepsilon - \frac{\varepsilon}{2\lambda_\mathrm{D}^2}\psi^2 \frac{\nabla n_0}{n_0} - \frac{\varepsilon}{\lambda_\mathrm{D}^2}\psi \boldsymbol{E}_0 \ . \tag{S32}$$

Solving Eq. (S24) for the force density in Eq. (S32) one obtains the slip velocity:

$$v_\parallel = \frac{1}{\eta}\int_0^\infty z \left(f_x - \frac{\mathrm{d}P}{\mathrm{d}x} \right) \mathrm{d}z = \frac{\varepsilon\zeta^2}{8\eta} \left(\frac{1}{T}\frac{\mathrm{d}T}{\mathrm{d}x} - \frac{1}{\varepsilon}\frac{\mathrm{d}\varepsilon}{\mathrm{d}x} - \frac{1}{n_0}\frac{\mathrm{d}n_0}{\mathrm{d}x} \right) - \frac{\varepsilon\zeta}{\eta} E_{0x} \ . \tag{S33}$$

If we neglect the permittivity and salinity gradients and no external electric field is applied ($E_0 = 0$) we find:

$$v_\parallel = \frac{\varepsilon\zeta^2}{8\eta}\frac{1}{T}\frac{\mathrm{d}T}{\mathrm{d}x} \ . \tag{S34}$$

In view of Eq. (S25) and (S26) the excess enthalpy of the electric-double layer contribution is given by:

$$h_\mathrm{E}(z) = -\frac{\varepsilon\zeta^2}{2\lambda_\mathrm{D}^2}\,\mathrm{e}^{-2z/\lambda_\mathrm{D}} \ , \tag{S35}$$

and for the thermo-osmotic coefficient we find:

$$\chi_\mathrm{E} = \frac{\varepsilon\zeta^2}{8\eta} \ . \tag{S36}$$

Van der Waals Contribution

The potential energy of a solvent molecule at distance z from a surface is

$$\varphi(z) = -\frac{A_\mathrm{H}}{3\pi z^3} V_0 \ , \tag{S37}$$

where A_H is the Hamaker constant and V_0 the volume of a solvent molecule. We note that the solvent concentration is given as $c_0 = 1/V_0$. The resulting force on the solvent molecules reads as:

$$\boldsymbol{f} = -c\,\nabla\varphi \ . \tag{S38}$$

Note that the gradient is normal to the surface and there is no force parallel to the surface, $f_x = 0$. The concentration may be taken constant in the normal direction, $f_z = -c\,\partial_z\varphi$, and integration of the normal component of the Stokes equation ($\partial_z P = f_z$) yields:

$$P = P_0 - c\varphi \ . \tag{S39}$$

We retain the concentration gradient parallel to the surface, $\partial_x P = -\varphi\,\partial_x c$ and evaluate:

$$v_\parallel = -\frac{1}{\eta}\int_{d_0}^\infty z \left(f_x - \frac{\mathrm{d}P}{\mathrm{d}x} \right) \mathrm{d}z = -\frac{1}{\eta}\int_{d_0}^\infty z\varphi \frac{\mathrm{d}c}{\mathrm{d}x}\,\mathrm{d}z = \frac{1}{\eta}\int_{d_0}^\infty \frac{A_\mathrm{H}}{3\pi z^2}\frac{1}{c_0}\frac{\mathrm{d}c}{\mathrm{d}x}\,\mathrm{d}z = -\frac{A_\mathrm{H}}{3\pi\eta d_0}\frac{1}{c_0}\frac{\mathrm{d}c}{\mathrm{d}x} = \frac{A_\mathrm{H}\beta}{3\pi\eta d_0}\frac{\mathrm{d}T}{\mathrm{d}x} \ , \tag{S40}$$

where we have used the relations:

$$\frac{\mathrm{d}c}{\mathrm{d}x} = \frac{\mathrm{d}c}{\mathrm{d}T}\frac{\mathrm{d}T}{\mathrm{d}x} \quad \text{and} \quad \beta = -\frac{1}{c_0}\frac{\mathrm{d}c}{\mathrm{d}T} \ , \tag{S41}$$

with the thermal expansion coefficient β. Note that the van der Waals potential diverges at $z = 0$, where the solvent molecules are in contact with the surface. To address this singularity we introduced a cut-off parameter d_0 corresponding to the size of a solvent molecule. In view of Eq. (S25) and (S26) the excess enthalpy of the van der Waals contribution is given by:

$$h_\mathrm{vdW}(z) = \frac{A_\mathrm{H}\beta T}{3\pi\eta z^3} \ , \tag{S42}$$

and for the thermo-osmotic coefficient we find:

$$\chi_\mathrm{vdW} = \frac{A_\mathrm{H}\beta T}{3\pi\eta d_0} \ . \tag{S43}$$

Numerical Simulation

To simulate the thermo-osmotic flow field we combined the heat transfer module (see Section 7 for details) with the laminar flow module. The sample geometry is the same as in Section 7 where the laminar flow module is defined only the fluid domain (Fig. S21, gray area).

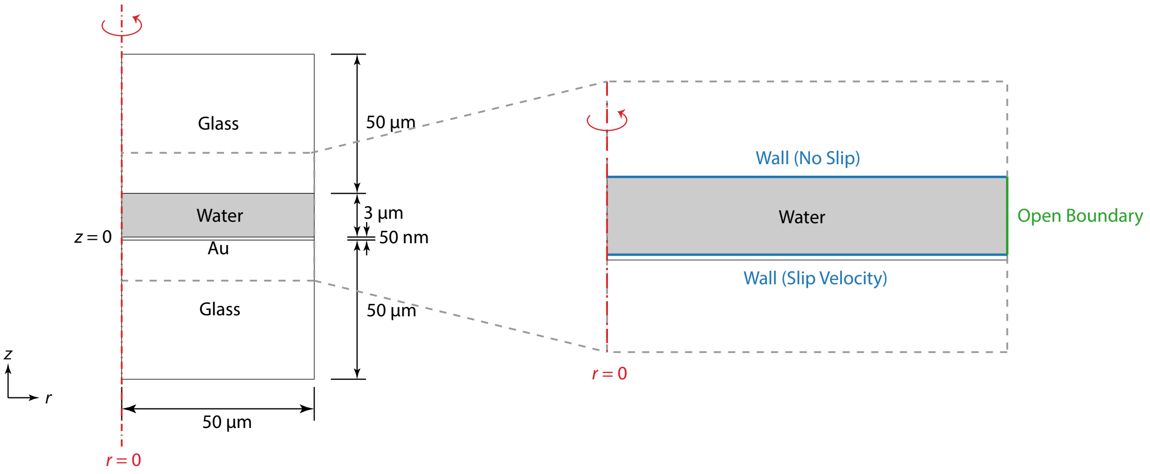

Fig. S21: Sketch of the 2D axisymmetric model employed to simulate the temperature distribution in the sample using COMSOL Multipyhsics 5.1 (dimensions are not to scale). The heat source domain, *i.e.*, the gold film, is colored in gray.

To account for the thermo-osmotic slip flow the lower boundary was defined as a wall with a slip velocity:

$$v_{\|} = \frac{\sigma_{\mathrm{T}}\,\eta}{\varrho}\frac{\nabla_{\|}T}{T}\ , \tag{S44}$$

where σ_{T} is a dimensionless thermal slip coefficient, η the viscosity and ϱ the density of the fluid. In case of water ($\eta = 0.001$ Pa s, $\varrho = 1000$ kg m^{-3}) the thermal slip coefficient σ_{T} is related to the thermo-osmotic coefficient χ with:

$$\sigma_{\mathrm{T}} = \chi \cdot 10^6\ \mathrm{m}^{-2}\,\mathrm{s}\ . \tag{S45}$$

In our simulation we have used $\chi = 10 \cdot 10^{-10}\ \mathrm{m}^2\,\mathrm{s}^{-1}$, *i.e.*, $\sigma_{\mathrm{T}} = 0.001$. The upper boundary was defined as Wall with no slip and the boundary on the right-hand side as open boundary.

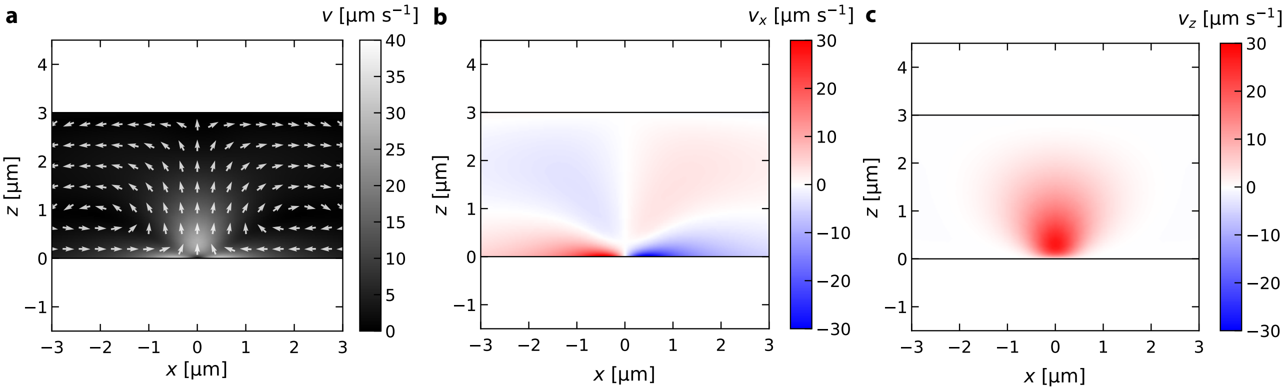

Fig. S22: Simulated thermo-osmotic flow field for an incident laser power of 1 mW. **a**, The magnitude of the flow velocity and its direction (arrows) in the xz-plane at $y = 0$. **b**, x-component of the flow velocity shown in (**a**). **c**, z-component of the flow velocity in (**a**).

Fig. S23 depicts the vorticity $\boldsymbol{\omega} = \nabla \times \boldsymbol{v}$ calculated from the flow field in Fig. S22. For a given vorticity $\boldsymbol{\omega}$ a sphere would rotate with a frequency of $\frac{1}{2}|\omega|$.

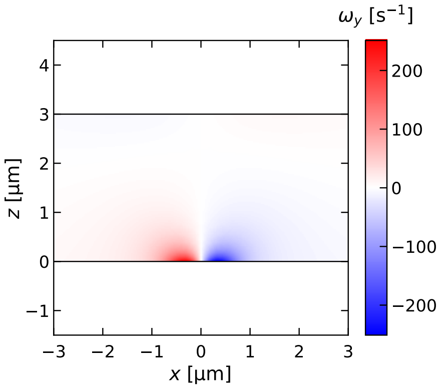

Fig. S23: The vorticity $\omega = \nabla \times v$ of the flow field in Fig. S22.

10 Thermophoresis of Colloidal Particles

In respect of Eq. (S24) only the force component parallel to the interface is relevant. Because of the different material properties of particle and solvent the applied temperature gradient is modified close to the interface. From the continuity condition of the heat flux we find:

$$\frac{\mathrm{d}T}{\mathrm{d}x} = \frac{3\kappa_1}{2\kappa_1 + \kappa_2} |\nabla T| \sin\theta \ , \tag{S46}$$

where κ_2 and κ_1 are the thermal conductivities of the particle and the surrounding liquid, respectively.

Dielectric Particles

For dielectric particles suspended in water we have $\kappa_1 \approx \kappa_2$ and $\mathrm{d}T/\mathrm{d}x \approx |\nabla T| \sin\theta$. From Eq. (S26) we find:

$$v_{\|}(\theta) = \chi \frac{|\nabla T|}{T} \sin\theta = v_{\|} \sin\theta \ . \tag{S47}$$

The slip velocity $v_{\|}$ varies along the particle surface with the polar angle as $\sin\theta$. The maximum value is observed at the midplane $\theta = \pi/2$.

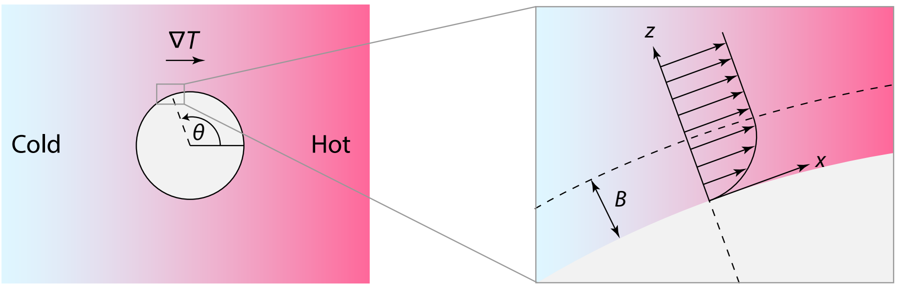

Fig. S24: Sketch of a spherical particle in a temperature gradient.

The particle velocity $\boldsymbol{v}$ is obtained from the orientational average of the slip velocity $v_{\|}(\theta)$ over the surface:

$$\boldsymbol{u} = -\langle v_{\|}(\theta)\, \boldsymbol{e}_x \rangle_\theta \tag{S48}$$

with the orientational average defined as:

$$\langle f(\theta)\rangle_\theta = \frac{\displaystyle\int_0^\pi f(\theta)\sin\theta\,\mathrm{d}\theta}{\displaystyle\int_0^\pi \sin\theta\,\mathrm{d}\theta} = \frac{1}{2}\int_0^\pi f(\theta)\sin\theta\,\mathrm{d}\theta \tag{S49}$$

In spherical coordinates the unit vector $\boldsymbol{e}_x$, parallel to the particle surface, is expressed as:

$$\boldsymbol{e}_x = \sin\theta\cos\varphi\,\boldsymbol{e}_r + \cos\theta\cos\varphi\,\boldsymbol{e}_\theta - \sin\theta\,\boldsymbol{e}_\varphi = \sin\theta\,\boldsymbol{e}_r + \cos\theta\,\boldsymbol{e}_\theta \ , \tag{S50}$$

with $\varphi = 0$. Inserting Eq. (S47) and (S50) in (S48) yields:

$$\boldsymbol{u} = -v_\parallel \langle \sin^2\theta\rangle_\theta\,\boldsymbol{e}_r - v_\parallel \langle \sin\theta\cos\theta\rangle_\theta\,\boldsymbol{e}_\theta \ . \tag{S51}$$

Using Eq. (S49) we find $\langle \sin^2\theta\rangle_\theta = 2/3$, $\langle \sin\theta\cos\theta\rangle_\theta = 0$ and hence:

$$\boldsymbol{u} = -\frac{2}{3} v_\parallel\,\boldsymbol{e}_r \ . \tag{S52}$$

Finally, with $v_\parallel$ defined in Eq. (S47), the thermophoretic particle velocity gets:

$$\boldsymbol{u}_\mathrm{T} = -\frac{2}{3}\chi\frac{\nabla T}{T} \ . \tag{S53}$$

With the thermophoretic mobility D_T defined as:

$$\boldsymbol{u}_\mathrm{T} = -D_\mathrm{T}\nabla T \tag{S54}$$

we find $D_\mathrm{T} = 2\chi/(3T)$ and $D_\mathrm{T} = \varepsilon\zeta^2/(12T)$ using Eq. (S36) for $\chi \approx \chi_\mathrm{E}$. For a dielectric particle with $\zeta \approx -40$ mV in water ($\varepsilon = 80\varepsilon_0$) we find $D_\mathrm{T} \approx 0.3\ \mu\mathrm{m}^2\,\mathrm{K}^{-1}\,\mathrm{s}^{-1}$, where we have used $T = 293.15$ K. For a temperature gradient in the order of $10\ \mathrm{K}\,\mu\mathrm{m}^{-1}$ (Fig. S16) we obtain particle velocities in the order of $u \approx 3\ \mu\mathrm{m}\,\mathrm{s}^{-1}$.

Metal Particles

In case of metal particles we have $\kappa_2 \gg \kappa_1$ and the particle velocity might be written as:

$$\boldsymbol{u}_\mathrm{T} = -\frac{2}{3}\chi\gamma\frac{\nabla T}{T} \ , \tag{S55}$$

with $\gamma = 3\kappa_1/(2\kappa_1 + \kappa_2)$ to account for the difference in the thermal conductivities. For a gold NP ($\kappa_2 = 318\ \mathrm{W\,m^{-1}\,K^{-1}}$) suspended in water ($\kappa_1 = 0.6\ \mathrm{W\,m^{-1}\,K^{-1}}$) we find $\gamma = 0.006$. This is about two orders of magnitude smaller than for dielectic particles ($\gamma \approx 1$). Due to their high thermal conductivity metal particles reduce the local temperature gradient leading to substantially smaller thermophoretic velocities. Fig. S25 compares the local temperature around a polystyrene (PS) and a AuNP subjected to an external temperature gradient of $1\ \mathrm{K}\,\mu\mathrm{m}^{-1}$. As analyzed in Fig. S26a, b the AuNP alters the local temperature (Fig. S26a) and quenches the temperature gradient on the particle surface (Fig. S26b).

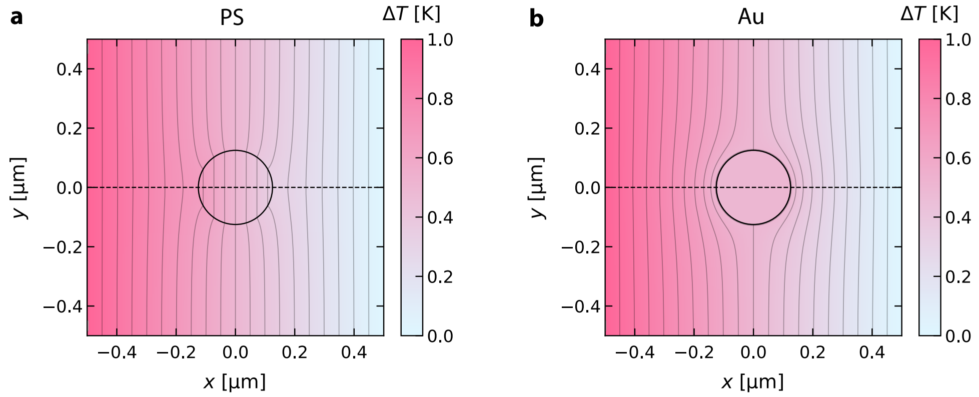

Fig. S25: The temperature distortion in the vicinity of a spherical particle in an external temperature gradient of $1\ \mathrm{K}\,\mu\mathrm{m}^{-1}$ for a PS NP (**a**) compared to a AuNP (**b**).

Fig. S26c depicts the particle velocity as function of the particle thermal conductivity κ_2 for $\chi = 1 \cdot 10^{-10}$ m^2 s^{-1} and $\kappa_1 = 0.6$ W m^{-1} K^{-1} (water). For $\kappa_2 \gg \kappa_1$ the particle velocity u scales with $u \propto \kappa_2^{-1}$. For AuNPs we find a thermometric velocity of 0.01 µm s^{-1} more than two orders of magnitude smaller than of dielectric particles. Hence, thermophoresis of AuNPs and metal NPs in general can be neglected.

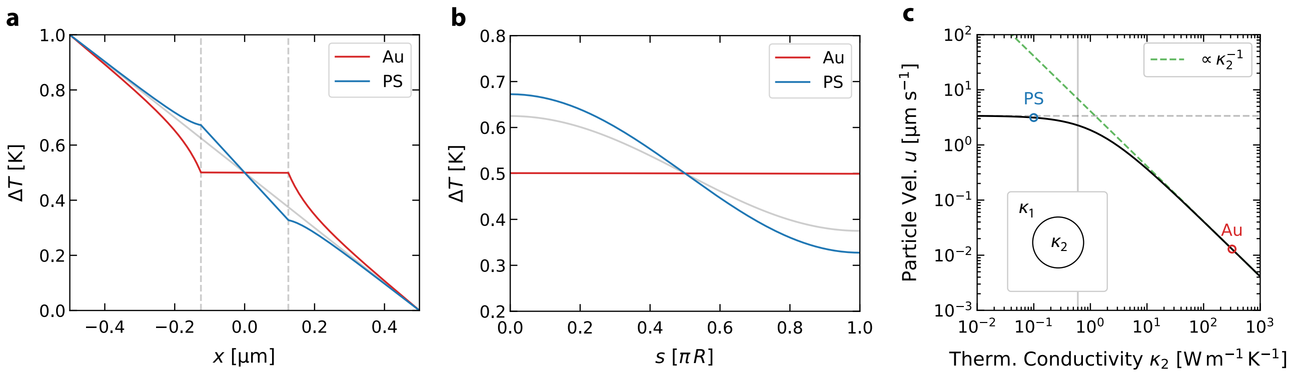

Fig. S26: **a**, Temperature on the dashed lines in Fig. S25. **b**, Temperature on the surface of the particles in Fig. S25. The gray lines in (**a**) and (**b**) depict the temperature profiles without a particle present. **c**, The particle velocity u as function of the particle thermal conductivity κ_2 for $\chi = 1 \cdot 10^{-10}$ m^2 s^{-1} and $\kappa_1 = 0.6$ W m^{-1} K^{-1} (water).

11 Estimation of the Lateral Trap Stiffness

To estimate the lateral trap stiffness the lateral velocity field v_x (Fig. S22b) is converted to a hydrodynamic force field using $F_x = 6\pi\eta R\gamma_{||}v_x$ (Fig. S27a), where $\gamma_{||}(z)$ is the correction factor for the friction coefficient parallel to the surface given by Eq. (S13). We consider the force close to the surface (Fig. S27a, black dash line) and estimate the trap stiffness from a linear fit in the range -0.25 µm $< x < 0.25$ µm (Fig. S27b, blue solid line).

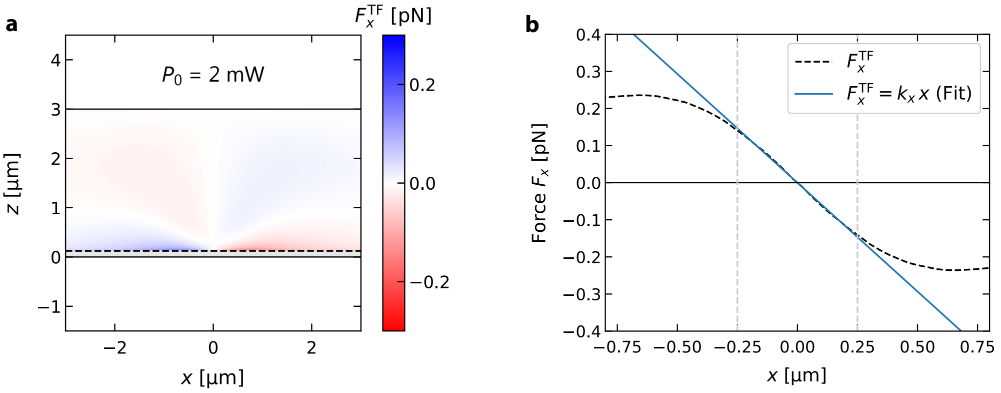

Fig. S27: **a**, The hydrodynamic force field for a AuNP with $R = 125$ nm radius at a heating power of $P_0 = 2$ mW. **b**, Fit of the force profile along the dash line in (**a**) with $F_x^{TF} = k_x x$ in the range -0.25 µm $< x < 0.25$ µm (blue solid line).

12 Optical Forces

For a spherical particle with radius $R \leq \lambda$ irradiated by an arbitrary electromagnetic wave the time-averaged radiation induced force can be approximated with:[14]

$$\langle \boldsymbol{F} \rangle = \frac{\varepsilon}{4} \operatorname{Re}(\alpha) \nabla |\boldsymbol{E}|^2 + \frac{\varepsilon}{2} \operatorname{Im}(\alpha) \operatorname{Im}\left(\sum_j E_j \nabla E_j^* \right), \tag{S56}$$

where the n runs over x, y, z and $\alpha(\omega) = \alpha_1(\omega) + \mathrm{i}\alpha_2(\omega)$ is the complex polarizability of the sphere. The total force can be split into two components; the gradient and the scattering/absorption force:

$$\langle \boldsymbol{F}_{\text{grad}} \rangle = \frac{\varepsilon}{4} \operatorname{Re}(\alpha) \, \nabla |\boldsymbol{E}|^2 \ , \tag{S57}$$

$$\langle \boldsymbol{F}_{\text{sca/abs}} \rangle = \frac{\varepsilon}{2} \operatorname{Im}(\alpha) \operatorname{Im}\left(\sum_j E_j \nabla E_j^* \right) . \tag{S58}$$

For a Gaussian beam the electric field $\boldsymbol{E}$ is given as:

$$E(r,z) = E_0 \frac{w_0}{w(z)} \exp\left(-\frac{r^2}{w(z)^2} \right) \exp\left(-\mathrm{i}kz - \mathrm{i}k \frac{r^2}{2R(z)} + \mathrm{i}\zeta(z) \right) \ , \tag{S59}$$

where $w(z) = w_0 \sqrt{1 + (z/z_\mathrm{R})^2}$ is the radius of the beam, $z_\mathrm{R} = \pi w_0^2 / \lambda$ the Rayleigh range, $R(z) = z\,(1 + (z_\mathrm{R}/z)^2)$ the curvature of the wavefronts and $\zeta(z) = \arctan(z/z_\mathrm{R})$ the Gouy phase shift. If we insert the electric field for a Gaussian beam in the equations for the gradient and scattering force we get for r- and z-components:[14,15]

$$F_r^{\text{grad}}(r,z) = -\frac{2\varepsilon}{\pi} \operatorname{Re}(\alpha) \, |E_0|^2 r \frac{w_0^2}{w(z)^4} \exp\left(-\frac{2r^2}{w(z)^2} \right) \ , \tag{S60}$$

$$F_z^{\text{grad}}(r,z) = -\frac{\varepsilon}{\pi} \operatorname{Re}(\alpha) \, |E_0|^2 z \frac{w_0^4}{z_\mathrm{R}^2} \left(\frac{1}{w(z)^4} - \frac{2r^2}{w(z)^6} \right) \exp\left(-\frac{2r^2}{w(z)^2} \right) \ , \tag{S61}$$

$$F_r^{\text{sca/abs}}(r,z) = \frac{\varepsilon}{\pi} \operatorname{Im}(\alpha) \, |E_0|^2 r \frac{w_0^2}{w(z)^2} \frac{k}{R(z)} \exp\left(-\frac{2r^2}{w(z)^2} \right) \ , \tag{S62}$$

$$F_z^{\text{sca/abs}}(r,z) = \frac{\varepsilon}{\pi} \operatorname{Im}(\alpha) \, |E_0|^2 \frac{w_0^2}{w(z)^2} \left(k \left(1 - \frac{r^2}{2} \frac{z^2 - z_\mathrm{R}^2}{(z^2 + z_\mathrm{R}^2)^2} \right) - \frac{w_0^2}{z_\mathrm{R} w(z)^2} \right) \exp\left(-\frac{2r^2}{w(z)^2} \right) \ . \tag{S63}$$

The electric field amplitude is given by $|E_0| = 2/w_0 \sqrt{Z_0 P_0 / \pi}$, where $Z_0 = \sqrt{\mu_0/\varepsilon_0}$ is the impedance of free space and P_0 the incident laser power. For small particles with $R \ll \lambda$ the complex polarizability is given by the approximation:

$$\alpha(\omega) = 3V \frac{\varepsilon_2(\omega) - \varepsilon_1}{\varepsilon_2(\omega) + 2\varepsilon_1} \ , \tag{S64}$$

where $V = 4/3\pi R^3$ is the volume of the sphere and $\varepsilon_2(\omega)$ and ε_1 are the permittivity of the particle and the surrounding medium, respectively. For larger particles with $R \approx \lambda$ the first order approximation of the complex polarizability is given by:[16]

$$\alpha(\omega) = \frac{1 - (1/10)(\varepsilon_2(\omega) + \varepsilon_1)\,\xi^2}{(1/3 + \varepsilon_1/(\varepsilon_2(\omega) - \varepsilon_1)) - (1/30)(\varepsilon_2(\omega) + 10\varepsilon_1)\,\xi^2 - \mathrm{i}4\pi^2 \varepsilon_1^{3/2} V/(3\lambda^3)} \ , \tag{S65}$$

where $\xi = 2\pi R/\lambda$ is a size parameter. Fig. S28a depicts the calculated z-component of the total force on a $R = 125$ nm AuNP in water as function of the NP position for laser power of $P_0 = 1$ mW. We have used $\lambda = 532$ nm for the a wavelength and a beam waist of $w_0 = 0.56$ µm.

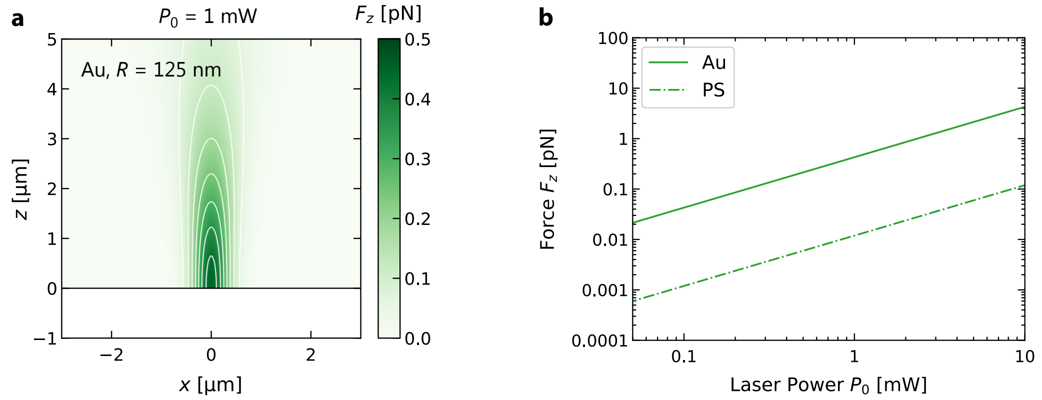

Fig. S28: **a**, The z-component of the total force on a $R = 125$ nm AuNP in water as function of the NP position for laser power of $P_0 = 1$ mW. We have used $\lambda = 532$ nm for the a wavelength and a beam waist of $w_0 = 0.56$ µm. **b**, The z-component of the total optical force at $z = 20$ nm as function of the incident laser power for a AuNP (solid line) and a PS NP (dash dotted line) of the same size.

For the given set of parameters the total force is mainly defined by the scattering force and the y-component can be neglected. The force as function of the incident laser power is depicted in Fig. S28b and compared between a Au and a PS NP of the same size. Due to their larger scattering cross-section the force on the AuNP is more than one order of magnitude larger than for a PS NP. Since only 10 % of the incident laser power is transmitted by the gold film (Fig. S14) the optical force in Fig. 3b of the main text was divided by a factor of 10.

13 Thermal Convection

To estimate the contribution of thermal convection we again combined the heat transfer module with the laminar flow module but in addition considered the temperature dependent density $\varrho(T)$ of the liquid. The simulation approach is similar to Fig. S21 where thermal convection was introduced by defining a force density $f_z = -\varrho(T)\, g$ within in the liquid sample domain. In contrast to Fig. S21 the lower and upper boundary have been defined as walls with no slip. Fig. S29 depicts the resulting temperature distribution and the relative density change within the sample. Note that the temperature distribution is essentially the same as in Fig. S15 and the effect of thermal convection on the temperature distribution can be neglected due to the small sample height.

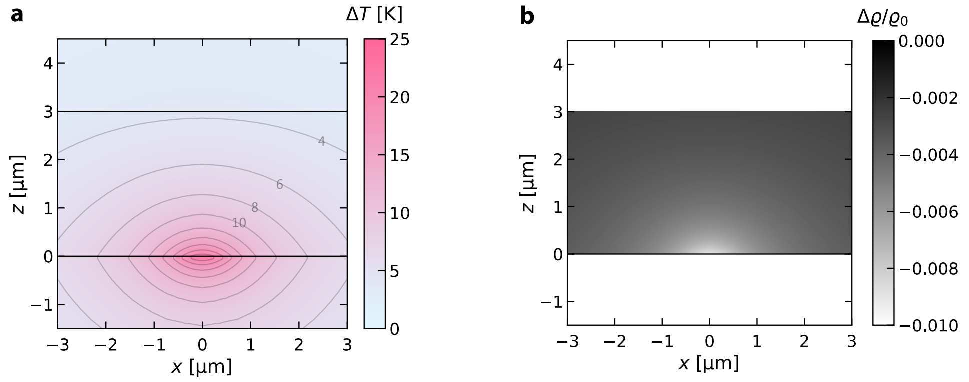

Fig. S29: Simulated temperature distribution and density change considering thermal convection. **a**, Temperature distribution in the xz-plane ($y = 0$). **b**, The relative density change in the xz-plane ($y = 0$).

The resulting convective flow field is depicted in Fig. S30. The expected flow velocities due to thermal convection are in the order of several $\mathrm{nm\,s^{-1}}$. This is four orders of magnitude smaller than the observed thermo-osmotic flow velocities (Fig. S22) Hence, the contribution of thermal convection is negligible for the given sample height (3 µm).

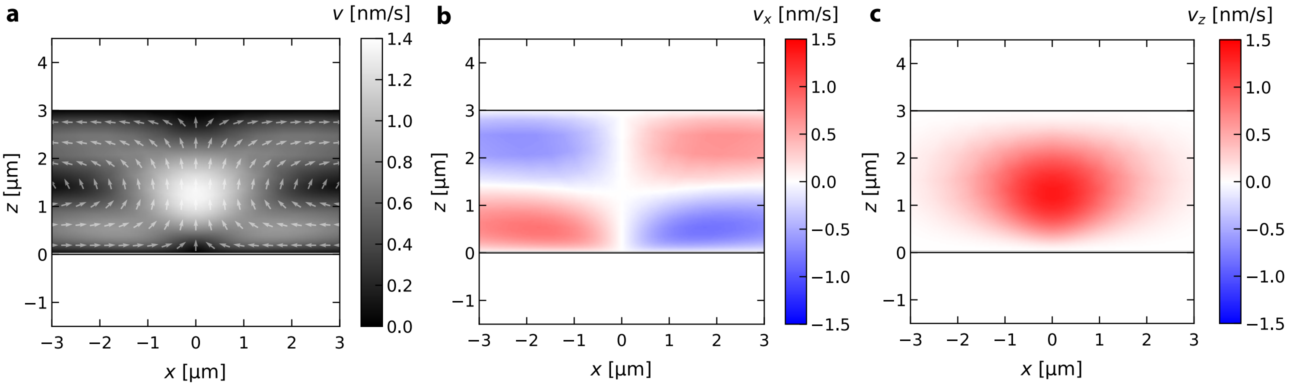

Fig. S30: Simulated flow field due to thermal convection for a laser power of 1 mW. **a**, Magnitude of the flow velocity and its direction (arrows) in the xz-plane ($y = 0$). **b**, x-component of the flow velocity shown in (**a**). **c**, z-component of the flow velocity in (**a**).

The maximum velocity $v_{\max}$ in the sample increases with the sample height (Fig. S31). Nevertheless, even for a sample height of $H = 80$ µm flow velocities well below 1 µm s^{-1} are expected. Notably, the maximum velocity close to the gold film ($z = 125$ nm) saturates with increasing sample height.[17]

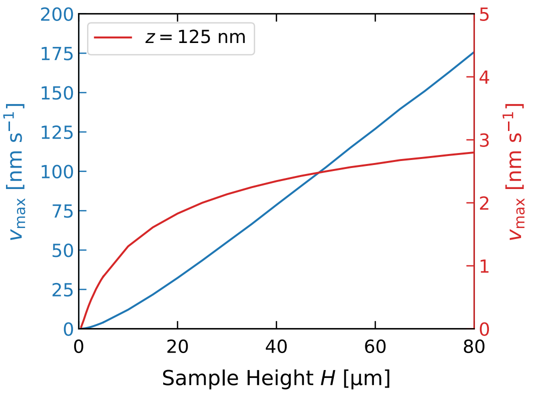

Fig. S31: The maximum velocity $v_{\max}$ due to thermal convection as function of the sample height H.

14 Depletion in Surfactant Solutions

Here, we consider the case of thermally driven depletion for particles larger than the gyration radius of the added surfactant molecules. The interaction potential φ between the particle and the surfactant molecules results in an excess pressure:[18]

$$P = n k_{\mathrm{B}} T \left(\mathrm{e}^{-\varphi/(k_{\mathrm{B}}T)} - 1\right) . \tag{S66}$$

Since the lateral component of the force is zero, $f_x = 0$, Eq. S24) reads:

$$v_{\parallel} = \frac{1}{\eta} \int_0^\infty z \left(f_x - \frac{\mathrm{d}P}{\mathrm{d}x} \right) \mathrm{d}z = -\frac{1}{\eta} \frac{\mathrm{d}}{\mathrm{d}x} n k_{\mathrm{B}} T \int_0^\infty z \left(\mathrm{e}^{-\varphi/(k_{\mathrm{B}}T)} - 1\right) \mathrm{d}z \tag{S67}$$

For an infinite repulsive potential with $\varphi = \infty$ for $z < 0$ and $\varphi = 0$ for $z > R$ the integral takes the value $\frac{1}{2}R^2$ and we find:

$$v_{\parallel} = -\frac{k_{\mathrm{B}}}{2\eta} R_{\mathrm{m}}^2 \frac{\mathrm{d}}{\mathrm{d}x} (nT) . \tag{S68}$$

and Eq. (S52) yields the transport velocity:

$$\boldsymbol{u}_{\mathrm{D}} = -\frac{k_{\mathrm{B}}}{3\eta} R_{\mathrm{m}}^2 \nabla (nT) . \tag{S69}$$

If we assume a molecular Soret coefficient S_T^m the surfactant concentration is given by $n(\boldsymbol{r}) = n_0 \exp(-S_T^m \Delta T(\boldsymbol{r}))$. The relative density distribution n/n_0 for the temperature distribution in Fig. S15 is plotted in Fig. S32, where we have used $S_T^m = 0.03\ \mathrm{K}^{-1}$.

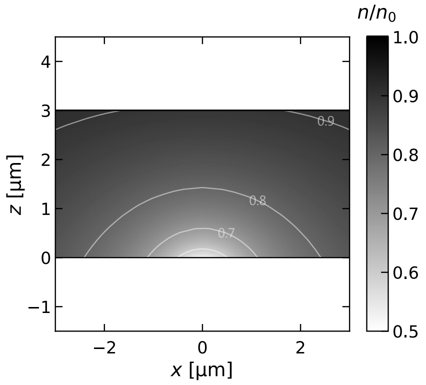

Fig. S32: The relative density distribution $n/n_0 = \exp(-S_T^m \Delta T(r))$ for the temperature distribution given in Fig. S15. Here, we have used $S_T^m = 0.03\ \mathrm{K}^{-1}$.

With $\nabla n = -n S_T^m \nabla T$ we find:

$$\boldsymbol{u}_D = -\frac{k_B}{3\eta} R_m^2 (\nabla n T + n \nabla T) = \frac{k_B}{3\eta} R_m^2 n (T S_T^m - 1) \nabla T \ . \tag{S70}$$

For the total transport velocity including thermophoresis we find:

$$\boldsymbol{u} = \boldsymbol{u}_T + \boldsymbol{u}_D = -\left(D_T - \frac{k_B}{3\eta} R_m^2 n (T S_T^m - 1) \right) \nabla T \ , \tag{S71}$$

with the thermophoretic mobility $D_T = 2\chi/(3T)$. In case of the surfactant sodiumdodecyl sulfate (SDS) we have $R_m = 2$ nm and $S_T^m = 0.03\ \mathrm{K}^{-1}$ and find $-0.43\ \mu\mathrm{m}^2\,\mathrm{K}^{-1}\,\mathrm{s}^{-1}$ for the depletion term on the right-hand side at a concentration of 5 mM. Hence, the depletion may easily exceed the thermophoretic mobility $D_T \approx 0.3\ \mu\mathrm{m}^2\,\mathrm{K}^{-1}\,\mathrm{s}^{-1}$ rendering the overall mobility negative, *i.e.*, the PS NPs are driven to the heated spot.[19]

Supplementary References

[1] J. C. Crocker and D. G. Grier, Methods of Digital Video Microscopy for Colloidal Studies, *J. Colloid Interface Sci.* **179**, 298–310 (1996).

[2] D. B. Allan, T. Caswell, N. C. Keim, and van der Wel, Casper M., *Trackpy v0.4.2*, Zenodo, Oct. 2019.

[3] M. Speidel, A. Jonáš, and E.-L. Florin, Three-Dimensional Tracking of Fluorescent Nanoparticles with Subnanometer Precision by Use of off-Focus Imaging, *Opt. Lett.* **28**, 69 (2003).

[4] J. N. Israelachvili, *Intermolecular and Surface Forces*, 3rd Edition (Elsevier, Academic Press, Amsterdam, 2011).

[5] H. Hamaker, The London-van Der Waals Attraction between Spherical Particles, *Physica* **4**, 1058–1072 (1937).

[6] M. Giesbers, J. Kleijn, and M. A. Cohen Stuart, The Electrical Double Layer on Gold Probed by Electrokinetic and Surface Force Measurements, *J. Colloid Interface Sci.* **248**, 88–95 (2002).

[7] J. Happel and H. Brenner, *Low Reynolds number hydrodynamics*, edited by R. J. Moreau, Vol. 1, Mechanics of Fluids and Transport Processes (Springer Netherlands, Dordrecht, 1981).

[8] H. Brenner, The Slow Motion of a Sphere through a Viscous Fluid towards a Plane Surface, *Chem. Eng. Sci.* **16**, 242–251 (1961).

[9] B. Lin, J. Yu, and S. A. Rice, Direct Measurements of Constrained Brownian Motion of an Isolated Sphere between Two Walls, *Phys. Rev. E* **62**, 3909–3919 (2000).

[10] P. B. Johnson and R. W. Christy, Optical Constants of the Noble Metals, *Phys. Rev. B* **6**, 4370–4379 (1972).

[11] G. Langer, J. Hartmann, and M. Reichling, Thermal Conductivity of Thin Metallic Films Measured by Photothermal Profile Analysis, *Rev. Sci. Instrum.* **68**, 1510–1513 (1997).

[12] R. Horn, Refractive Indices and Order Parameters of Two Liquid Crystals, *J. Phys. France* **39**, 105–109 (1978).

[13] M. Marinelli, F. Mercuri, U. Zammit, and F. Scudieri, Thermal Conductivity and Thermal Diffusivity of the Cyanobiphenyl (nCB) Homologous Series, *Phys. Rev. E* **58**, 5860–5866 (1998).

[14] A. S. Urban, S. Carretero-Palacios, A. A. Lutich, T. Lohmüller, J. Feldmann *et al.*, Optical Trapping and Manipulation of Plasmonic Nanoparticles: Fundamentals, Applications, and Perspectives, *Nanoscale* **6**, 4458 (2014).

[15] R. R. Agayan, F. Gittes, R. Kopelman, and C. F. Schmidt, Optical Trapping near Resonance Absorption, *Appl. Opt.* **41**, 2318 (2002).

[16] H. Kuwata, H. Tamaru, K. Esumi, and K. Miyano, Resonant Light Scattering from Metal Nanoparticles: Practical Analysis beyond Rayleigh Approximation, *Appl. Phys. Lett.* **83**, 4625–4627 (2003).

[17] J. S. Donner, G. Baffou, D. McCloskey, and R. Quidant, Plasmon-Assisted Optofluidics, *ACS Nano* **5**, 5457–5462 (2011).

[18] A. Würger, Thermal Non-Equilibrium Transport in Colloids, *Rep. Prog. Phys.* **73**, 126601 (2010).

[19] D. Vigolo, S. Buzzaccaro, and R. Piazza, Thermophoresis and Thermoelectricity in Surfactant Solutions, *Langmuir* **26**, 7792–7801 (2010).
