# Supplementary material for: Hydrodynamic manipulation of nano-objects by optically induced thermo-osmotic flows
Source: Nat Commun. 2022 Feb 3;13:656. doi: 10.1038/s41467-022-28212-z (PMC8813924; doi:10.1038/s41467-022-28212-z)
Supplement: Supplementary file 3 — Description of additional Supplementary File [file 41467_2022_28212_MOESM3_ESM.pdf]

Descriptions of additional Supplementary data files

Supplementary Movie 1

Free diffusion of AuNPs over an Au film in DI water (left) and in 10 mM NaCl (right) without heating. The AuNPs have a radius of 125 nm.

Supplementary Movie 2

AuNP over a locally heated Au film in DI water for a laser power of 2 mW (left) and the detected particle size and location (right). The AuNP has a radius of 125 nm.

Supplementary Movie 3

AuNP over a locally heated Au film in DI water (left) and 10 mM NaCl (right) for a laser power of 1.25 mW. The AuNP has a radius of 125 nm.

Supplementary Movie 4

Trapped AuNP over a locally heated Au film in 10 mM NaCl for four different laser powers. The AuNP has a radius of 125 nm.

Supplementary Movie 5

Trapped AuNP with 50 nm radius over a heated Au film in 30 mM NaCl for a laser power of 1.0 mW.

Supplementary Movie 6

Two AuNPs trapped at different distances using a multiplex, focused laser beam with a laser power of 2 mW. The AuNPs have a radius of 125 nm.

Supplementary Movie 7

Three AuNPs trapped in a triangular pattern using a multiplex, focused laser beam with a laser power of 3 mW. The AuNPs have a radius of 125 nm.

Supplementary Movie 8

Trapped AuNP dynamically manipulated to follow a circular path. The AuNP has a radius of 125 nm.

Supplementary Movie 9

AuNPs driven by thermo-viscous and thermo-osmotic flows. The laser beam scans a circular path at high frequency (f = 0.5 kHz) in clockwise direction with a laser power of 10 mW.

Supplementary Movie 10

Single AuNP in an ensemble of PS NPs over a locally heated Au film in 5 mM SDS for a laser power of 1.5 mW. The AuNP as well as the PS NPs have a radius of 125 nm.

Supplementary Movie 11

Single AuNP in an ensemble of PS NPs (R = 250 nm) over a locally heated Au film in 10 mM NaCl for a laser power of 1 mW. The AuNP as well as the PS NPs have a radius of 125 nm.

Supplementary Movie 12

Three PS ellipsoids (2.39 μm major-axis length, 0.34 μm minor-axis length) over a locally heated Au film in 5 mM SDS for a laser power of 1.5 mW.
